# Genetic Susceptibility to Impaired Lung Function in Underground Coal Miners from Kazakhstan

**DOI:** 10.3390/genes17091021

**Published:** 2026-08-27

**Authors:** Anastassiya Perfilyeva, Sayagul Kairgeldina, Alexandr Gulyayev, Kanat Tekebayev, Kanagat Yergali, Ozada Khamdiyeva, Sholpan Koigel’dinova, Zhanbol Sabirov, Nazym Sagandykova, Madina Baurzhan, Gulnur Zhunussova

**Affiliations:** 1 Laboratory of Molecular Genetics, Institute of Genetics and Physiology, Almaty 050060, Kazakhstan; 2 Scientific Research Institute of Balneology and Medical Rehabilitation of the Ministry of Health of the Republic of Kazakhstan, Astana 010000, Kazakhstan; 3 National Laboratory Astana, Nazarbayev University, Astana 010000, Kazakhstan; 4 Department of Clinical Pharmacology and Evidence-Based Medicine, NC JSC “Karaganda Medical University”, Karaganda 100030, Kazakhstan; 5 Laboratory of Experimental Mutagenesis, Institute of Genetics and Physiology, Almaty 050060, Kazakhstan; 6 Department of Internal Medicine, NC JSC “Karaganda Medical University”, Karaganda 100030, Kazakhstan; 7 NC JSC National Centre for Occupational Health and Diseases, Karaganda 100008, Kazakhstan; 8 Corporate Fund “University Medical Center”, Astana 010000, Kazakhstan; doctor.ent.alm@gmail.com

**Keywords:** coal miners, occupational dust exposure, lung function, spirometry, genetic susceptibility, Kazakhstan

## Abstract

Background/Objectives: Occupational exposure to coal mine dust is a major risk factor for respiratory impairment; however, the genetic determinants of individual variation in lung function remain poorly understood, particularly in occupational cohorts that have been largely absent from genome-wide association studies. This study aimed to identify genetic variants associated with quantitative lung function traits in underground coal miners from Kazakhstan. Methods: A total of 186 underground coal miners from the Karaganda coal basin were genotyped using the Illumina Global Screening Array. Genome-wide association analyses were performed for six spirometric parameters (FVC, FEV1, FEV1/FVC, MEF25, MEF50, and MEF75), adjusting for age, mining exposure, body mass index, smoking status, and population structure. Genotype imputation was performed to increase genomic coverage and evaluate the consistency of association signals. Results: No variants reached the conventional genome-wide significance threshold. However, recurrent suggestive associations identified *PRKCB*, *FANCE*, and *UBTD1* as the principal candidate loci across correlated spirometric parameters, with independent support from genotype imputation. Conclusions: Recurrent associations across complementary spirometric parameters identified biologically plausible candidate loci involved in inflammatory signalling, DNA repair, and epithelial homeostasis. These findings provide one of the first genomic insights into lung function in underground coal miners and provide a foundation for future validation and functional studies.

## 1. Introduction

Lung function is a fundamental indicator of respiratory health and a strong predictor of chronic respiratory disease, disability, and premature mortality [1,2,3]. Spirometric indices, including forced vital capacity (FVC), forced expiratory volume in one second (FEV1), the FEV1/FVC ratio, and maximal expiratory flow indices (MEF25, MEF50, and MEF75), provide quantitative measures of pulmonary function and are widely used to detect airflow limitation, characterize obstructive ventilatory defects, and identify early small-airway dysfunction [4,5]. Impaired lung function frequently precedes the clinical onset of chronic respiratory diseases and results from the complex interplay between environmental exposures and inherited genetic factors [6,7,8].

Among environmental determinants, occupational exposure is one of the strongest contributors to accelerated lung function decline. Underground coal miners represent one of the occupational groups at highest risk because of prolonged exposure to respirable coal mine dust, crystalline silica, diesel exhaust particles, and other airborne contaminants generated during underground mining operations [9,10,11,12]. These exposures induce persistent airway inflammation, oxidative stress, epithelial injury, and tissue remodelling, ultimately contributing to airflow limitation and chronic respiratory impairment. Epidemiological studies have consistently reported increased prevalence of chronic respiratory disease among miners compared with non-mining populations [13,14,15,16,17]. Nevertheless, miners with comparable occupational exposures often exhibit substantial inter-individual variability in respiratory outcomes, suggesting that environmental factors alone cannot fully explain susceptibility to impaired lung function.

Genetic factors contribute substantially to normal variation in lung function. Twin and family studies have estimated the heritability of spirometric parameters to be approximately 40–60%, indicating that inherited variation plays a major role in determining pulmonary function [18,19,20]. Consistent with these observations, genome-wide association studies (GWAS) have established lung function as a highly polygenic trait, identifying hundreds of genetic loci involved in lung development, epithelial differentiation, extracellular matrix organization, inflammation, airway remodelling, and tissue repair [21,22,23]. As GWAS sample sizes have increased and more ancestrally diverse populations have been included, the number of associated loci has expanded substantially. The largest multi-ancestry GWAS to date, involving nearly 600,000 individuals, reported more than 1,000 independent signals associated with spirometric parameters and chronic obstructive pulmonary disease (COPD) susceptibility [24]. Population-specific studies have shown that, although many loci are shared across ancestries, additional ancestry-specific associations also contribute to lung function variability, confirming the importance of conducting genetic studies in underrepresented populations [25,26,27,28,29,30]. To the best of our knowledge, the genetic architecture of lung function remains poorly characterised in Central Asia.

In addition, genetic studies of respiratory phenotypes in occupationally exposed workers remain relatively limited, particularly among coal miners. Most genetic studies have focused on susceptibility to coal workers’ pneumoconiosis (CWP) and silicosis, employing candidate-gene, genome-wide, and transcriptome-wide approaches to identify susceptibility loci and genes associated with these occupational lung diseases [31,32,33]. Nevertheless, these studies have focused predominantly on disease susceptibility rather than quantitative lung function phenotypes. Compared with dichotomous disease phenotypes, quantitative spirometric parameters capture the full spectrum of pulmonary function and may identify early biological alterations preceding clinically overt respiratory disease [6,8].

In the present study, we investigated the genetic determinants of lung function in underground coal miners from Kazakhstan to identify genetic loci associated with quantitative spirometric parameters in an occupational cohort exposed to coal mine dust.

## 2. Materials and Methods

### 2.1. Study Population and Data Collection

Participants were recruited in 2025 from underground coal mines in the Karaganda coal basin (Kirovskaya, Kuzembaeva, Saranskaya, Abayskaya, Lenina, Shakhtinskaya, and Tentekskaya) located in the Karaganda Region of Central Kazakhstan. A total of 186 underground coal miners were enrolled in the study. Spirometric, demographic, and occupational data were collected from all participants.

Spirometry was performed by trained personnel using a BTL-08 Spiro spirometer (BTL Industries Ltd., Stevenage, UK) in accordance with the American Thoracic Society/European Respiratory Society (ATS/ERS) standards [5] following bronchodilator administration with 400 μg salbutamol (Salbutamol-AV, 100 μg/dose; Altayvitaminy CJSC, Biysk, Russia). The evaluated parameters included forced vital capacity (FVC), forced expiratory volume in one second (FEV1), the FEV1/FVC ratio, and maximal expiratory flow at 25%, 50%, and 75% of forced vital capacity (MEF25, MEF50, and MEF75).

Demographic and occupational information was obtained using a structured questionnaire, including age, ethnicity, smoking status, body height, body weight, years of underground mining experience, and occupation. The diagnosis of chronic bronchitis associated with occupational dust exposure was established by an expert occupational pathology commission of the National Center for Occupational Hygiene and Occupational Diseases (Karaganda, Kazakhstan). The diagnosis was based on clinical symptoms and history (cough for more than 3 months per year for at least two consecutive years, sputum production, dyspnoea, and healthcare utilisation due to chronic bronchitis), together with outpatient and inpatient medical records, including results of mandatory pre-employment and periodic medical examinations, as well as laboratory and functional investigations. Chronic bronchitis status was used to characterise the clinical composition of the cohort; genetic association analyses were focused on quantitative spirometric traits across the entire cohort rather than on a binary clinical diagnosis. Information on a history of bronchial asthma, rhinitis, and allergy was obtained by participant self-report. Smoking status was classified as never, current, or former smoker. Body mass index (BMI) was calculated as weight (kg) divided by height squared (m^2^). Occupational categories were harmonised into standardised categories, including face workers, drift miners, underground electricians, mining equipment operators, mining technicians/fitters, mine foremen/supervisors, rescue and safety personnel, support staff, and surveying/engineering personnel.

Peripheral blood samples were transported to the Laboratory of Molecular Genetics, Institute of Genetics and Physiology (Almaty, Kazakhstan), under controlled temperature conditions in portable cool boxes and stored at −20 °C until DNA extraction.

### 2.2. DNA Extraction and Genotyping

Genomic DNA was extracted from whole blood using the ReliaPrep™ Blood gDNA Miniprep System (Promega, Madison, WI, USA) according to the manufacturer’s protocol. DNA concentration and purity were assessed using a NanoDrop OneC spectrophotometer (Thermo Fisher Scientific, Waltham, MA, USA).

Genome-wide genotyping was performed using the Illumina Global Screening Array-24 v3.0 BeadChip (Illumina, San Diego, CA, USA). The array contains approximately 654,000 markers, including 85,342 exonic and 262,173 intronic markers, as well as variants located in untranslated, promoter, and splice regions [34]. The mean and median inter-marker spacing are approximately 4.4 and 2.3 kb, respectively. Thus, the array provides broad representation of both coding and non-coding genomic regions. Arrays were scanned using the iScan System (Illumina, San Diego, CA, USA), and genotype calling and initial quality control were performed using GenomeStudio software v2.0.4 (Illumina, San Diego, CA, USA).

### 2.3. Genotype Quality Control and Population Structure

Raw genotype data were converted to PLINK binary format and merged across genotyping batches. Only autosomal variants (chromosomes 1–22) were retained for downstream analyses, and duplicate variants mapping to identical genomic positions were removed. Quality control was performed using PLINK v2.0 [35]. Samples with genotype call rates < 98% were excluded. Variants were filtered based on missingness (>2%), minor allele frequency (MAF < 1%), and Hardy–Weinberg equilibrium in controls (*p* < 1 × 10^−6^). Related individuals were identified by pairwise identity-by-descent (IBD) analysis. For pairs with estimated relatedness (PI_HAT > 0.1875), one individual from each pair was removed.

Population structure was assessed by principal component analysis (PCA). Prior to PCA, linkage disequilibrium (LD) pruning was performed using a sliding-window approach (200 kb window, 50 variants per step, r^2^ < 0.2). The first five principal components (PC1–PC5) were included as covariates in all association analyses.

### 2.4. Genotype Imputation

Following quality control, genotypes were exported in chromosome-specific VCF v4.2 format using PLINK v2.0 [35]. Variant coordinates were reported relative to the GRCh37/hg19 reference genome, and alleles were aligned to the forward strand according to the Illumina GSA-24v3-0_A1 Strand Report. Genotype imputation was performed using the TOPMed Imputation Server pipeline v2.0.13-statgen.1 with the TOPMed reference panel and Eagle v2.4 for haplotype phasing [36]. Imputed variants with an imputation quality score (Rsq) < 0.8 or MAF < 1% were excluded from downstream analyses.

### 2.5. Genome-Wide Association and Statistical Analyses

Pearson correlation coefficients among the six spirometric parameters (FVC, FEV1, FEV1/FVC ratio, MEF25, MEF50, and MEF75) were calculated in R version 4.4.2 (R Foundation for Statistical Computing, Vienna, Austria) using the cor() function with pairwise complete observations. Correlation matrices were visualised as heatmaps using the corrplot package v0.95 [37].

Genome-wide association analyses were performed separately for directly genotyped variants and imputed genotype dosages under an additive genetic model using PLINK v2.0 [35]. The six spirometric parameters (FVC, FEV1, FEV1/FVC ratio, MEF25, MEF50, and MEF75) were analysed as continuous outcomes using linear regression. Models were adjusted for age, mining experience, BMI, smoking status, and the first five genetic principal components (PC1–PC5). Effect sizes (β coefficients), standard errors, and two-sided *p* values were estimated for each variant.

Multiple testing was considered using the conventional genome-wide significance threshold (*p* < 5 × 10^−8^). Suggestive associations were defined as *p* < 1 × 10^−5^. Quantile–quantile (QQ) and Manhattan plots were generated using the qqman package v0.1.9 in R version 4.4.2 [38] to assess the distribution of association *p* values. The genomic inflation factor (λ_GC_) was calculated to evaluate residual population stratification and inflation of test statistics.

### 2.6. Variant Annotation

Variants were annotated for genomic location and predicted functional consequences using the Ensembl Variant Effect Predictor (VEP) web interface based on the GRCh37/hg19 reference genome [39]. Gene mapping and previously reported associations with respiratory phenotypes were evaluated using publicly available resources, including the GWAS Catalog [40].

## 3. Results

### 3.1. Characteristics of the Study Cohort

The study cohort comprised 186 underground coal miners recruited from seven underground coal mines in the Karaganda coal basin. Baseline characteristics of the overall cohort are summarised in Table 1, while detailed participant-level demographic, occupational, clinical, and spirometric data are provided in Table S1.

### 3.2. Spirometric Parameter Correlations

**Figure 1:**
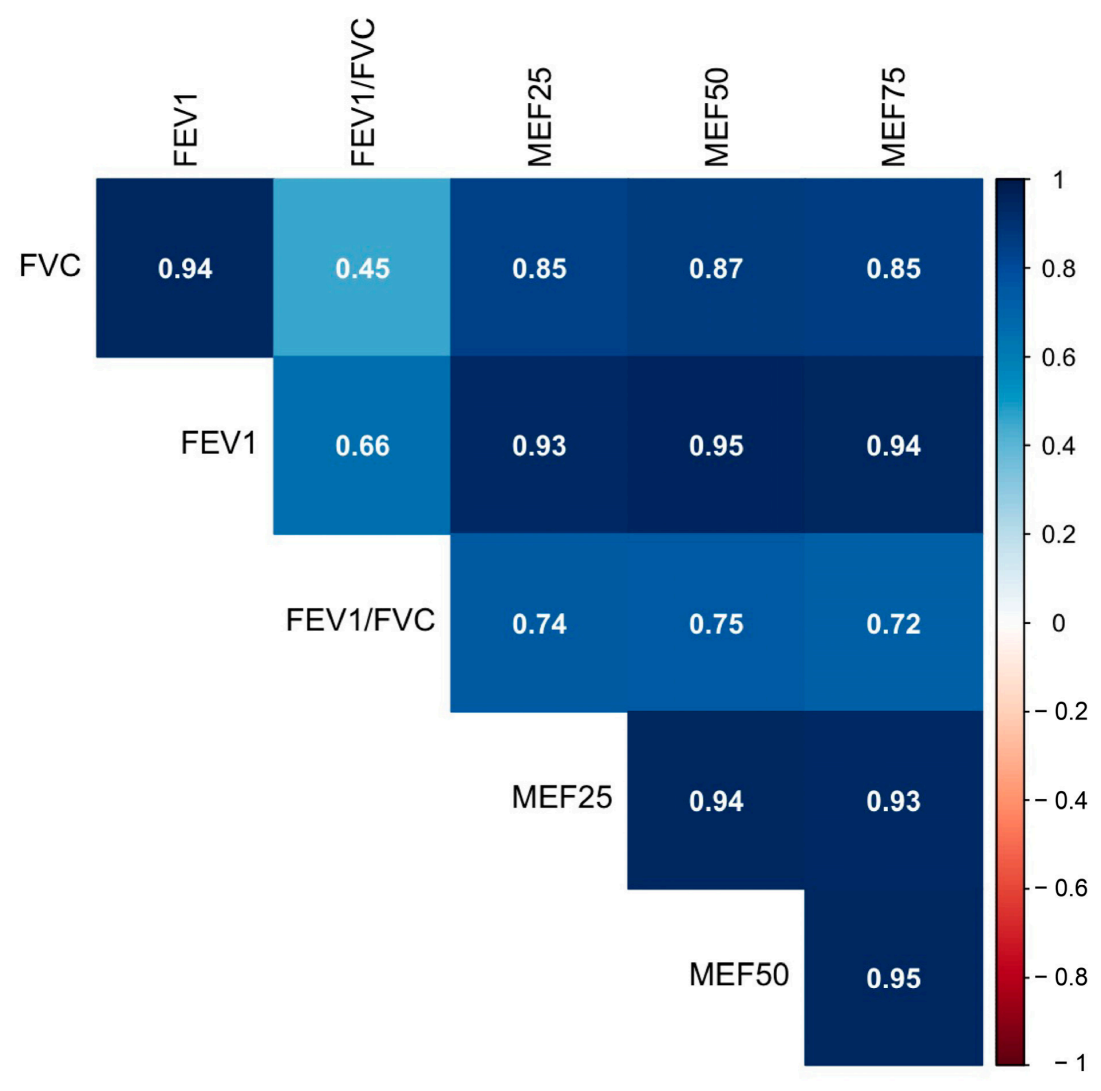
Pairwise correlations between spirometric parameters in the study cohort.

Pairwise correlations among the six spirometric parameters are shown in Figure 1. Strong positive correlations were observed across all spirometric parameters, with the highest correlations between FEV1 and FVC (r = 0.94), between FEV1 and the maximal expiratory flow parameters (MEF25–MEF75; r = 0.93–0.95), and among the three MEF indices (r = 0.93–0.95). In contrast, the FEV1/FVC ratio showed only moderate correlations with FVC (r = 0.45), FEV1 (r = 0.66), and the maximal expiratory flow parameters (r = 0.72–0.75).

### 3.3. Genotyping Quality Control

The initial genotype dataset comprised 186 individuals and 650,181 genotyped variants. After restricting the dataset to autosomal chromosomes, 616,843 variants remained. Removal of variants mapping to identical genomic positions resulted in 614,660 SNPs. Quality control filtering based on genotype missingness and MAF retained 459,110 variants. Exclusion of variants deviating from Hardy–Weinberg equilibrium (*p* < 1 × 10^−6^) yielded a final dataset of 459,091 variants. The final dataset included 186 individuals and 459,091 variants for downstream analyses. The overall genotype call rate after quality control exceeded 99%.

For population structure analysis, LD pruning yielded 186,768 approximately independent variants, which were used to compute principal components.

### 3.4. Population Structure

**Figure 2:**
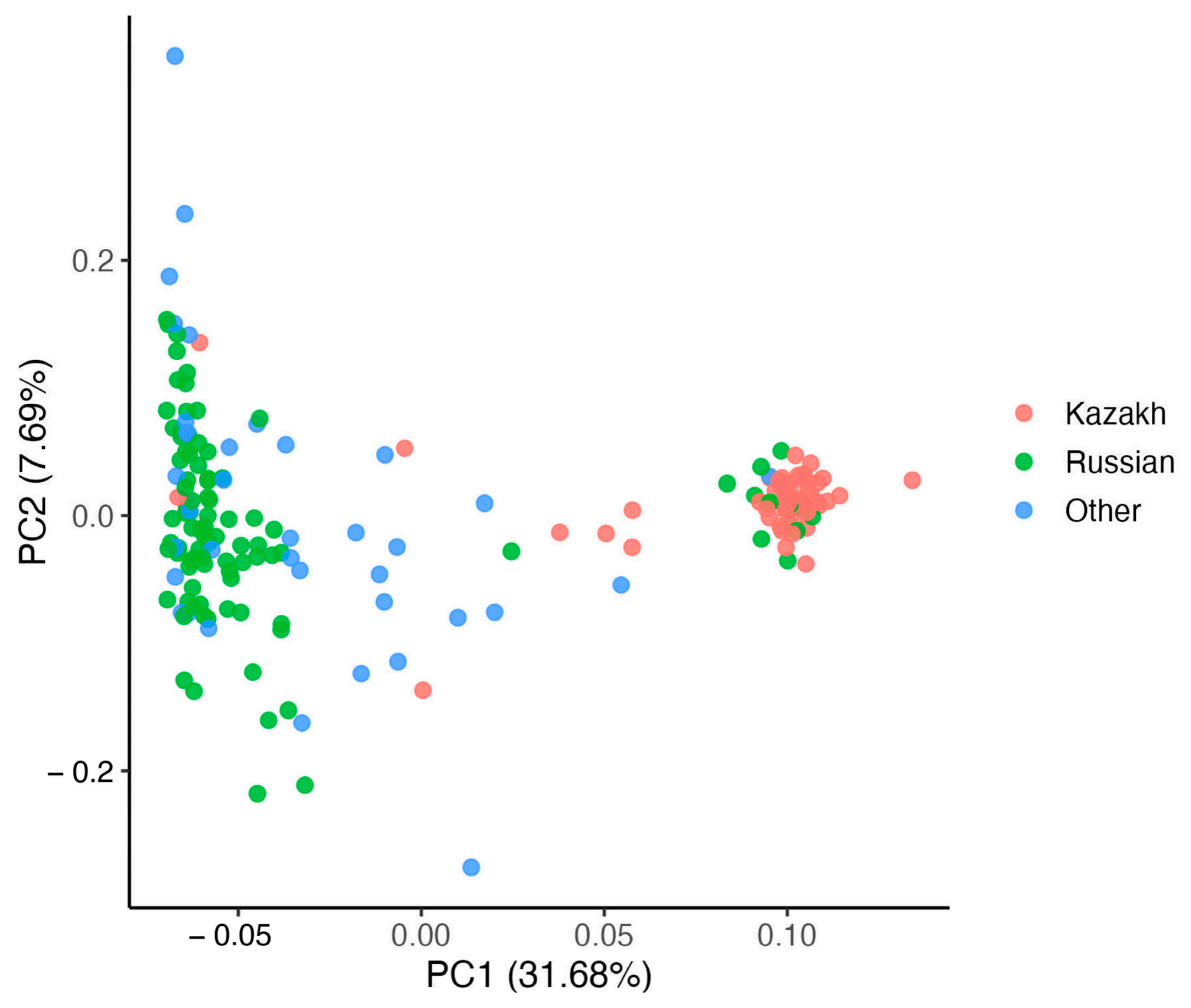
Population structure of the study cohort. Scatter plot of the first two principal components (PC1 and PC2) derived from LD-pruned autosomal variants (n = 186,768 SNPs) in the unrelated study sample (n = 186). Points represent individual participants and are coloured according to self-reported ethnicity.

PCA was performed to assess genetic population structure in the study cohort. The first two principal components explained 31.68% and 7.69% of the total genetic variance, respectively (Figure 2). The PCA plot demonstrated clear population structure, with two major clusters broadly corresponding to self-reported ethnicity and partial overlap between them, consistent with the admixed composition of the cohort. To minimise potential confounding due to population stratification, the first five principal components were included as covariates in all subsequent association analyses.

### 3.5. Genome-Wide Association Analyses of Lung Function Traits

**Figure 3:**
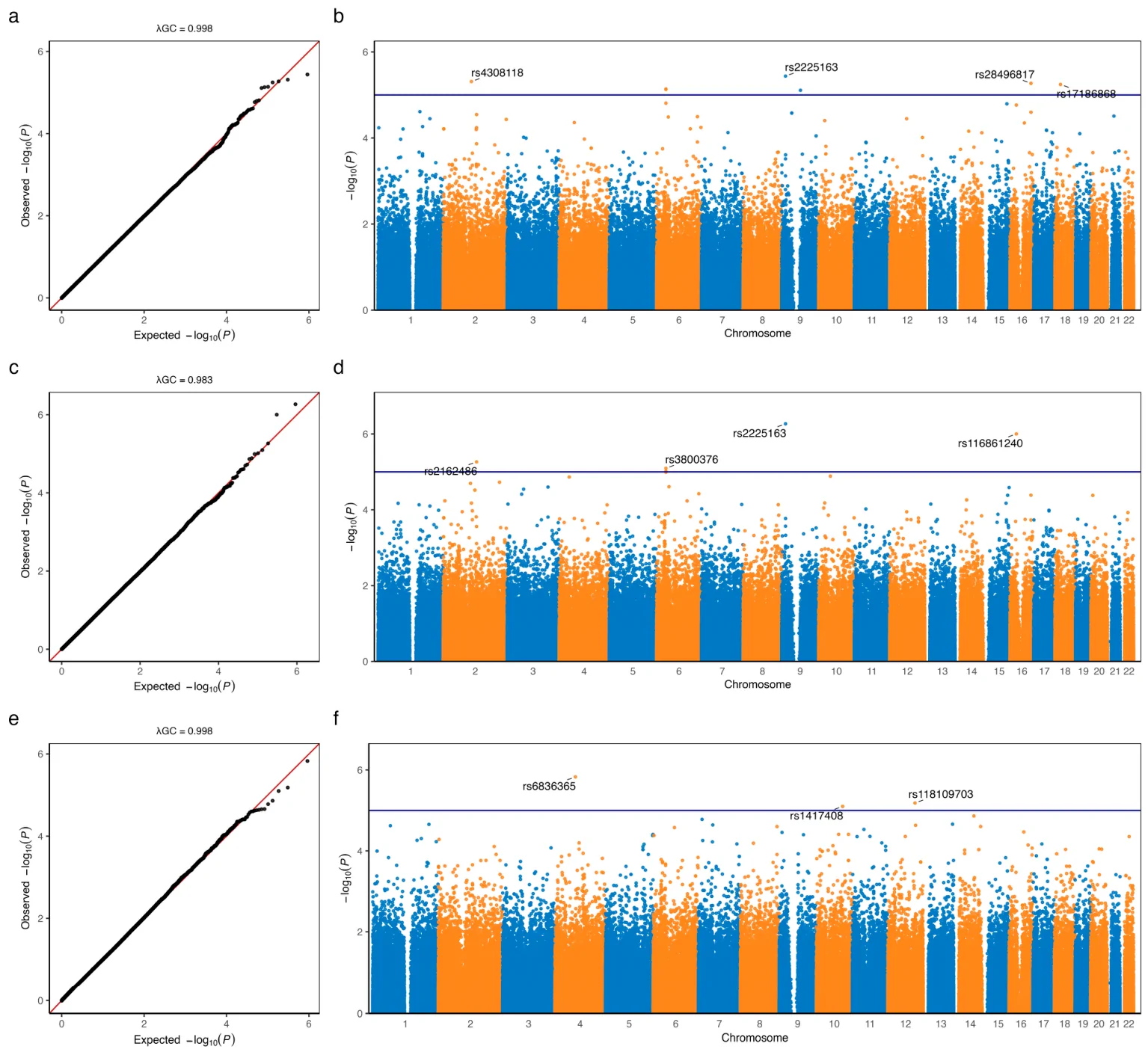
Genome-wide association analysis of major spirometric parameters using directly genotyped variants: (**a**) QQ plot of observed versus expected *p* values for FVC. (**b**) Manhattan plot showing the distribution of association signals across autosomal chromosomes for FVC. (**c**) QQ plot of observed versus expected *p* values for FEV1. (**d**) Manhattan plot showing the distribution of association signals across autosomal chromosomes for FEV1. (**e**) QQ plot of observed versus expected *p* values for the FEV1/FVC ratio. (**f**) Manhattan plot showing the distribution of association signals across autosomal chromosomes for the FEV1/FVC ratio. In the QQ plots, black points represent the observed distribution of −log_10_(*p*) values, and the red diagonal line represents the expected distribution under the null hypothesis. λGC denotes the genomic inflation factor. In the Manhattan plots, blue and orange points distinguish variants on alternating chromosomes. The horizontal blue line indicates the suggestive significance threshold (*p* = 1 × 10^−5^), and selected variants exceeding this threshold are labelled.

**Figure 4:**
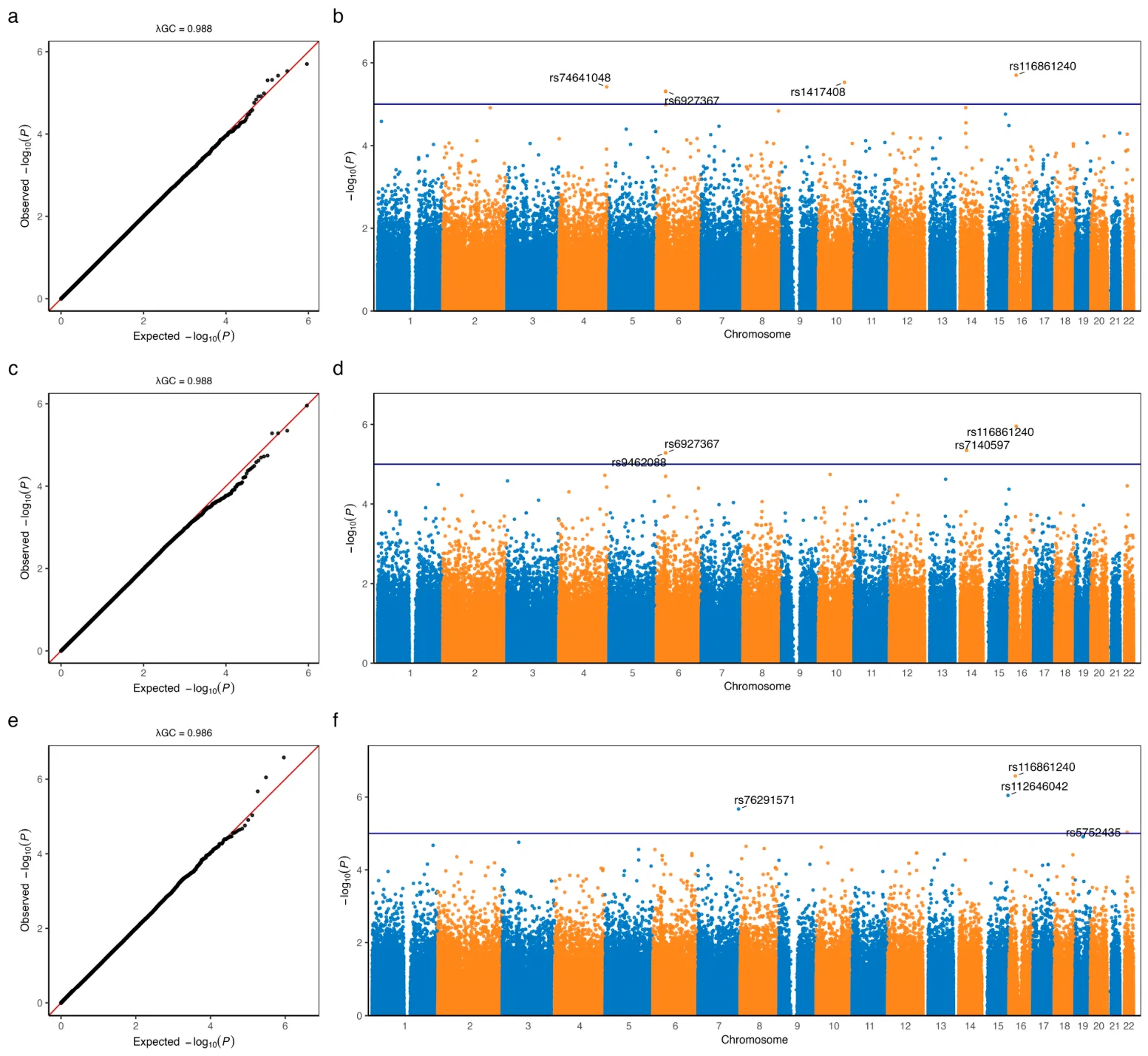
Genome-wide association analysis of small-airway function traits using directly genotyped variants: (**a**) QQ plot of observed versus expected *p* values for MEF25. (**b**) Manhattan plot showing the distribution of association signals across autosomal chromosomes for MEF25. (**c**) QQ plot of observed versus expected *p* values for MEF50. (**d**) Manhattan plot showing the distribution of association signals across autosomal chromosomes for MEF50. (**e**) QQ plot of observed versus expected *p* values for MEF75. (**f**) Manhattan plot showing the distribution of association signals across autosomal chromosomes for MEF75. In the QQ plots, black points represent the observed distribution of −log_10_(*p*) values, and the red diagonal line represents the expected distribution under the null hypothesis. λGC denotes the genomic inflation factor. In the Manhattan plots, blue and orange points distinguish variants on alternating chromosomes. The horizontal blue line indicates the suggestive significance threshold (*p* = 1 × 10^−5^), and selected variants exceeding this threshold are labelled.

**Figure 5:**
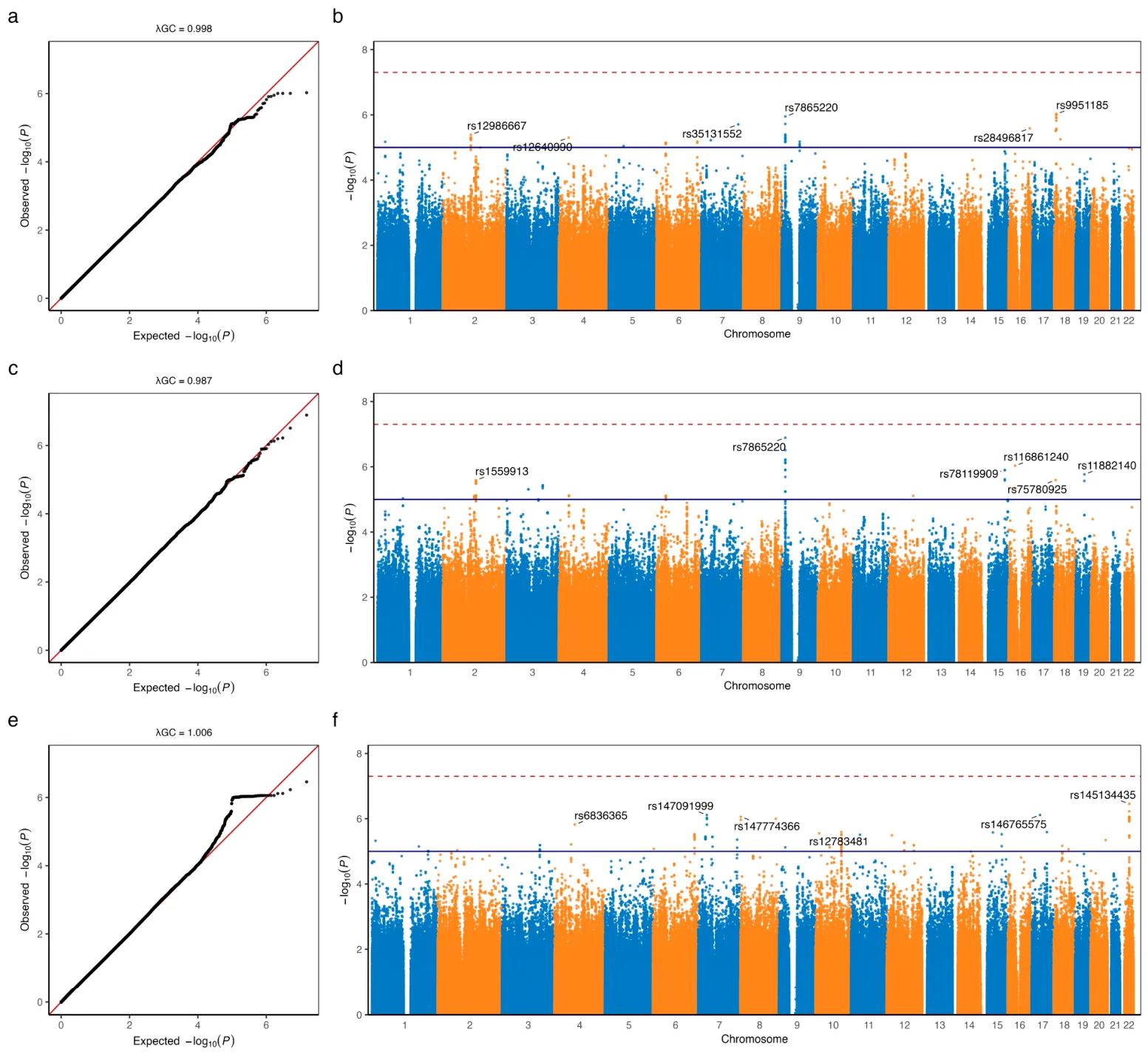
Genome-wide association analysis of major spirometric parameters following genotype imputation: (**a**) QQ plot of observed versus expected *p* values for FVC. (**b**) Manhattan plot showing the distribution of association signals across autosomal chromosomes for FVC. (**c**) QQ plot of observed versus expected *p* values for FEV1. (**d**) Manhattan plot showing the distribution of association signals across autosomal chromosomes for FEV1. (**e**) QQ plot of observed versus expected *p* values for the FEV1/FVC ratio. (**f**) Manhattan plot showing the distribution of association signals across autosomal chromosomes for the FEV1/FVC ratio. In the QQ plots, black points represent the observed distribution of −log_10_(*p*) values, and the red diagonal line represents the expected distribution under the null hypothesis. λGC denotes the genomic inflation factor. In the Manhattan plots, blue and orange points distinguish variants on alternating chromosomes. The horizontal blue line indicates the suggestive significance threshold (*p* = 1 × 10^−5^), and selected variants exceeding this threshold are labelled.

**Figure 6:**
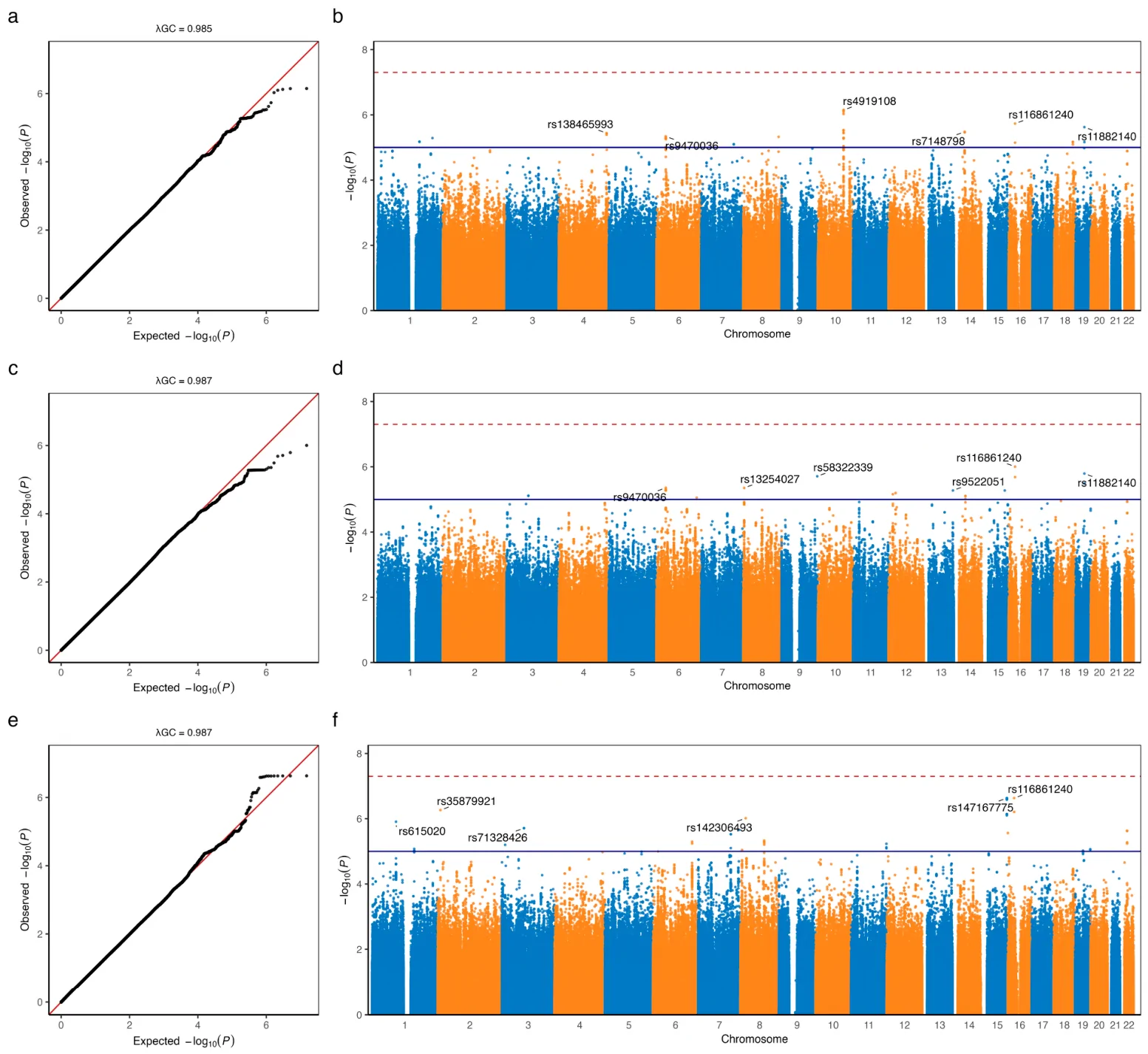
Genome-wide association analysis of small-airway function traits following genotype imputation: (**a**) QQ plot of observed versus expected *p* values for MEF25. (**b**) Manhattan plot showing the distribution of association signals across autosomal chromosomes for MEF25. (**c**) QQ plot of observed versus expected *p* values for MEF50. (**d**) Manhattan plot showing the distribution of association signals across autosomal chromosomes for MEF50. (**e**) QQ plot of observed versus expected *p* values for MEF75. (**f**) Manhattan plot showing the distribution of association signals across autosomal chromosomes for MEF75. In the QQ plots, black points represent the observed distribution of −log_10_(*p*) values, and the red diagonal line represents the expected distribution under the null hypothesis. λGC denotes the genomic inflation factor. In the Manhattan plots, blue and orange points distinguish variants on alternating chromosomes. The horizontal blue line indicates the suggestive significance threshold (*p* = 1 × 10^−5^), and selected variants exceeding this threshold are labelled.

Genome-wide association analyses were performed for six correlated spirometric parameters using both directly genotyped variants and imputed genotype dosages. QQ plots demonstrated well-controlled test statistics across all analyses, with λGC ranging from 0.983 to 0.998 for directly genotyped variants and from 0.985 to 1.006 following genotype imputation, indicating no evidence of residual population stratification or systematic inflation (Figure 3, Figure 4, Figure 5 and Figure 6). No variants reached the conventional genome-wide significance threshold (*p* < 5 × 10^−8^). However, multiple suggestive loci (*p* < 1 × 10^−5^) were identified, including several loci that demonstrated recurrent associations across multiple correlated lung function traits (Table 2).

The strongest cross-trait association signals were observed at the *PRKCB* locus, where the intronic variant rs116861240 was associated with FEV1, MEF25, MEF50, and MEF75. Recurrent associations were also detected at the *FANCE* locus, with rs9462088 associated with FVC, FEV1, MEF25, and MEF50, whereas rs6927367 was associated with FVC, MEF25, and MEF50. Additional loci showing repeated evidence across traits included *RP11-408A13.1* (rs2225163), associated with both FVC and FEV1, and *UBTD1* (rs1417408), associated with the FEV1/FVC ratio and MEF25.

Beyond these recurrent loci, additional trait-specific suggestive associations were identified for individual spirometric parameters (Figure 3 and Figure 4; Table S2). These included *RP11-345M22.1* (rs28496817) and *CABLES1* (rs17186868), together with several intergenic loci for FVC; *SHROOM3* (rs6836365) for the FEV1/FVC ratio; *AC108142.1* (rs74641048) for MEF25; and additional intergenic loci associated with MEF50 and MEF75.

Following genotype imputation, genome-wide association analyses were repeated using genotype dosages for 7,579,392 autosomal variants. Imputation substantially increased the number of suggestive association signals while supporting the principal loci identified using directly genotyped variants, including *PRKCB*, *FANCE*, *UBTD1*, *SHROOM3*, and *CABLES1* (Table 3; Figure 5 and Figure 6).

In addition, genotype imputation identified several additional suggestive loci that were not prioritised among the lead signals in the directly genotyped analysis. These included *ZDHHC21* (rs7865220), associated with both FVC and FEV1; *CHST8* (rs11882140), associated with FEV1, MEF25, and MEF50; *AGBL1* (rs78119909 and rs9708220), associated with FEV1 and MEF50; *CSMD1* (rs13254027), associated with MEF50; and *CSGALNACT1* (rs142306493) and *AXIN1* (rs577184980), associated with MEF75. The complete list of lead suggestive association signals identified following genotype imputation is provided in Table S3.

## 4. Discussion

In this exploratory genome-wide association study of underground coal miners from Kazakhstan, no variants reached the conventional genome-wide significance threshold; however, several loci showed consistent suggestive associations across multiple spirometric traits. Notably, *PRKCB*, *FANCE*, and *UBTD1* were each associated with two or more correlated lung function parameters and remained supported following genotype imputation, strengthening the robustness of these signals. Because spirometric traits reflect complementary aspects of pulmonary physiology, the repeated identification of the same loci across independent association analyses suggests that these genes may contribute to shared biological mechanisms underlying lung function. Although these findings require replication in larger independent cohorts, they provide new insights into the genetic architecture of lung function in underground coal miners and establish a foundation for future validation studies in occupationally exposed populations.

Among the prioritised loci, *PRKCB* represented the strongest candidate gene, with the intronic variant rs116861240 demonstrating recurrent suggestive associations with FEV1, MEF25, MEF50 and MEF75. *PRKCB* encodes protein kinase C β, a serine/threonine kinase that regulates multiple signalling pathways involved in immune activation, oxidative stress, apoptosis and inflammatory responses. Experimental studies have shown that activation of PKC signalling in human bronchial epithelial cells induces NF-κB- and TNF-α-dependent inflammatory responses, promotes IL-6 and IL-8 production, and contributes to epithelial injury [41]. Experimental studies further demonstrated that activation of PKCβ in bronchial epithelial cells exposed to cigarette smoke extract promoted mitochondrial dysfunction through phosphorylation of p66Shc, resulting in oxidative stress and epithelial injury [42]. Beyond experimental evidence, *PRKCB* has recently been implicated in susceptibility to pulmonary non-tuberculous mycobacterial disease, where increased gene expression enhances intracellular survival of *Mycobacterium avium* within macrophages [43], and altered *PRKCB* expression has also been associated with non-small cell lung cancer progression [44]. Collectively, these observations support the biological plausibility of *PRKCB* as a candidate gene influencing lung function in individuals chronically exposed to coal mine dust.

*FANCE* represented another recurrent locus, with variants showing suggestive associations across multiple spirometric parameters, including FVC, FEV1, MEF25 and MEF50. *FANCE* encodes a core component of the Fanconi anaemia DNA-repair pathway, which maintains genomic stability through the repair of DNA interstrand crosslinks. Chronic exposure to coal mine dust generates oxidative stress and DNA damage in airway epithelial cells, making efficient DNA-repair mechanisms essential for maintaining epithelial integrity. Experimental disruption of the Fanconi anaemia pathway results in chromosomal instability and impaired cellular responses to environmental carcinogens through suppression of the key effector protein FANCD2 [45]. In addition, pathway-based genetic analyses have implicated Fanconi anaemia genes in susceptibility to lung adenocarcinoma, further supporting the importance of this DNA-repair network in pulmonary tissues exposed to genotoxic environmental agents [46]. Given the role of FANCE in the FA DNA-repair pathway, genetic variation within *FANCE* may influence the ability of airway epithelial cells to respond to genotoxic stress induced by chronic occupational dust exposure.

The third recurrent locus identified in the present study was *UBTD1*, where rs1417408 was associated with both the FEV1/FVC ratio and MEF25. *UBTD1* encodes a ubiquitin domain-containing protein involved in ubiquitin-dependent protein turnover through interactions with members of the UBE2D family. Functional studies have demonstrated that UBTD1 regulates epidermal growth factor receptor (EGFR) signalling by controlling ceramide metabolism, endolysosomal positioning, receptor phosphorylation and lysosomal degradation [47]. In addition, UBTD1 influences protein stability through ubiquitin-dependent mechanisms and has been implicated in the regulation of cellular metabolism and proliferation via pathways involving c-Myc signalling [48]. Although *UBTD1* has not previously been implicated directly in occupational respiratory disease, its regulation of EGFR signalling is of particular biological interest because EGFR is a key mediator of airway epithelial responses to inhaled particulate matter. Experimental studies have shown that dust particles activate EGFR signalling, promoting epithelial inflammatory responses, tissue repair and airway remodelling following epithelial injury [49,50,51]. By modulating EGFR activation and receptor turnover, UBTD1 may therefore influence epithelial homeostasis and the pulmonary response to chronic occupational dust exposure, providing a biologically plausible mechanism linking this locus to variation in lung function.

Together, these findings suggest that genetic variation affecting inflammatory signalling, DNA-damage responses, and epithelial homeostasis may contribute to inter-individual differences in lung function among underground coal miners chronically exposed to coal mine dust. Although speculative, the convergence of independent association signals on these complementary biological processes strengthens the biological plausibility of the identified candidate loci.

The genetic findings of the present study complement a substantial body of previous research on lung function impairment in coal miners, which has largely focused on the effects of occupational dust exposure rather than the genetic determinants of quantitative lung function. Longitudinal studies of British coal miners demonstrated that FEV1 decline was associated with cumulative respirable dust exposure [52,53]. Comparable findings were reported in US coal miners, where longitudinal analyses demonstrated an association between occupational exposure and FEV1 decline [54], while a large cross-sectional study of 7139 miners found inverse associations of estimated cumulative dust exposure with FEV1, FVC, and FEV1/FVC, including among miners without radiographically detected CWP [55]. Accelerated FEV1 decline in underground coal miners has also been associated with air trapping and evidence suggestive of small-airway dysfunction, supporting the potential involvement of the peripheral airways in dust-related functional impairment [56]. Genetic studies in coal miners, in contrast, have predominantly focused on susceptibility to CWP and related disease phenotypes rather than genome-wide associations with quantitative spirometric traits. A GWAS of Han Chinese coal workers identified rs73329476 at 12q15 as a genome-wide significant susceptibility variant for silica-related pneumoconiosis, with the risk allele also associated with reduced expression of carboxypeptidase M (CPM) [57]. A subsequent analysis of subthreshold signals from this GWAS, incorporating genotype imputation and functional annotation, identified additional CWP-associated eQTL variants linked to *ATP1A4*, *FNBP1P1*, *ALS2CR12*, and *RBM26* [31]. The 12q15 region was also investigated functionally, with rs12812500 in *CPM* subsequently associated with silicosis susceptibility and altered *CPM* expression [58]. A different approach was taken by Wang et al., who examined whether variants previously associated with respiratory traits were also associated with CWP. They reported associations with variants linked to *FAM13A*, *ANGPTL1*, *SPATS2L*, and *RP11-463O9.9*, while Mendelian randomisation analysis suggested that genetically predicted higher lung function was associated with a lower risk of CWP [59]. Thus, previous research in coal miners has provided substantial evidence for both dust-related deterioration of lung function and genetic susceptibility to CWP, whereas the genetic basis of quantitative spirometric variation in this occupational population remains comparatively underexplored.

The absence of overlap between the principal loci identified in the present study and previously reported susceptibility loci for CWP or other fibrotic lung diseases may, at least in part, reflect differences in the phenotypes being investigated. Persistent dust-induced lung injury can lead to fibrotic remodelling of the lung parenchyma in susceptible individuals [60], and large genetic studies of pulmonary fibrosis have identified susceptibility loci involving genes such as *MUC5B*, *TERT*, *DSP*, *FAM13A*, *DPP9*, and *TOLLIP* [61,62]. Experimental studies have also implicated additional regulators of fibrotic responses, including the long non-coding RNA *FENDRR*, whose altered expression has been linked to fibroblast activation and fibrosis [63,64,65]. None of these previously implicated genes or loci emerged among the principal suggestive signals in the present study. However, CWP and pulmonary fibrosis are disease phenotypes defined by clinical and structural abnormalities, whereas the present analysis examined common genetic variation associated with quantitative measures of pulmonary function across the study cohort. CWP and other fibrotic lung diseases can substantially affect spirometric indices, including FVC and FEV1, but spirometry characterises physiological impairment rather than the presence or extent of structural lung disease, which is primarily assessed by thoracic imaging and, in selected settings, histopathological evaluation [66]. Accordingly, loci influencing quantitative variation in lung function need not coincide with loci conferring susceptibility to CWP or pulmonary fibrosis. The modest statistical power of the present exploratory cohort may have further limited the detection of previously reported disease-associated loci.

This study has several strengths. First, it addresses an important gap in genetic research by providing one of the first genome-wide analyses of quantitative lung function traits in underground coal miners. Second, the relatively homogeneous occupational environment of underground coal miners reduced heterogeneity arising from environmental exposures. Third, the analysis focused on multiple quantitative spirometric parameters rather than a single binary phenotype, enabling a more comprehensive assessment of the genetic architecture of lung function.

Several limitations should be considered when interpreting the present findings. First, the relatively modest sample size substantially limited statistical power and precluded the detection of genome-wide significant associations. Consequently, all identified loci should be regarded as hypothesis-generating and require validation in larger independent cohorts. Second, although genotype imputation substantially increased genomic coverage and provided convergent support for several recurrent loci, the findings have not yet been replicated in an independent study population. Third, despite adjustment for age, cumulative mining exposure, body mass index, smoking status, and population structure, residual confounding cannot be completely excluded because of differences in age and occupational exposure between participants. Fourth, pre-employment spirometric data were not available for the study participants, and the cross-sectional study design therefore precluded assessment of individual changes in lung function from pre-exposure baseline or longitudinal rates of lung function decline.

## 5. Conclusions

In conclusion, this exploratory genome-wide association study provides one of the first genome-wide investigations of quantitative lung function traits in underground coal miners. Although no variants reached genome-wide significance, recurrent associations across multiple correlated spirometric parameters identified *PRKCB*, *FANCE*, and *UBTD1* as the principal candidate loci influencing lung function, with additional support provided by genotype imputation. These findings suggest that inflammatory signalling, DNA repair, and epithelial homeostasis may contribute to inter-individual variation in pulmonary function among workers chronically exposed to coal mine dust. Further validation in larger occupational cohorts and functional studies will be essential to confirm these candidate loci and clarify their biological roles.

## Figures and Tables

**Table 1:** Characteristics of the study cohort.

Characteristics	Overall Cohort (*n* = 186)	Range
Age (years)	46.1 ± 11.7	27–75
Sex, *n* (%)		
Male	186 (100)	
Mining experience (years)	17.0 ± 11.1	0.3–43.0
BMI (kg/m^2^)	28.2 ± 4.1	18.8–42.4
Smoking status, *n* (%)		
Never smoker	124 (66.7)	
Current smoker	42 (22.6)	
Former smoker	20 (10.8)	
Ethnicity, *n* (%)		
Kazakh	53 (28.5)	
Russian	95 (51.1)	
Other	38 (20.4)	
Profession, *n* (%)		
Drift miner	26 (14.0)	
Face worker	33 (17.7)	
Mine foreman/supervisor	32 (17.2)	
Mining equipment operator	14 (7.5)	
Mining technician/fitter	45 (24.2)	
Underground electrician	27 (14.5)	
Rescue/safety personnel	2 (1.1)	
Support staff	6 (3.2)	
Surveying/engineering	1 (0.5)	
Clinical conditions, *n* (%)		
Chronic bronchitis	89 (47.8)	
Bronchial asthma	0 (0)	
Rhinitis	0 (0)	
History of allergy	0 (0)	
Spirometric parameter		
FVC (% predicted)	80.8 ± 24.5	35.6–133.0
FEV1 (% predicted)	84.5 ± 30.7	25.3–140.3
FEV1/FVC ratio (% predicted)	105.9 ± 13.2	63.4–127.7
MEF25 (% predicted)	80.6 ± 31.5	19.0–134.3
MEF50 (% predicted)	74.0 ± 30.1	14.6–128.6
MEF75 (% predicted)	67.2 ± 30.5	16.7–116.6

**Table 2:** Recurrent suggestive loci (*p* < 1 × 10^−5^) identified across multiple spirometric parameters using directly genotyped variants.

Gene/Locus	Gene/Locus Function	Variant(s)	Spirometric Parameters	Functional Annotation	Minimum *p* Value
*PRKCB*	Serine/threonine protein kinase involved in diverse cellular signalling pathways	rs116861240	FEV1, MEF25, MEF50, MEF75	intron variant	2.628 × 10^−7^
*FANCE*	Component of the Fanconi anaemia nuclear complex involved in DNA repair	rs9462088	FVC, FEV1, MEF25, MEF50	missense variant	4.967 × 10^−6^
		rs6927367	FVC, MEF25, MEF50	intron variant	4.859 × 10^−6^
*RP11-408A13.1*	Non-coding locus; function not well characterised	rs2225163	FVC, FEV1	upstream gene variant	5.394 × 10^−7^
*UBTD1*	Ubiquitin-family protein involved in regulation of UBE2D E2 ubiquitin-conjugating enzymes	rs1417408	FEV1/FVC ratio, MEF25	intron variant	2.970 × 10^−6^

**Table 3:** Recurrent and additional suggestive loci (*p* < 1 × 10^−5^) identified following genotype imputation.

Gene/Locus	Lead Variant(s)	Associated Spirometric Parameters	Status
*PRKCB*	rs116861240	FEV1, MEF25, MEF50, MEF75	Supported locus
*FANCE*	rs9470036, rs9470027	FVC, FEV1, MEF25, MEF50	Supported locus
*UBTD1*	rs12783481, rs4919108	FEV1/FVC ratio, MEF25	Supported locus
*SHROOM3*	rs6836365	FEV1/FVC ratio	Supported locus
*CABLES1*	rs17186868	FVC	Supported locus
*ZDHHC21*	rs7865220	FVC, FEV1	Additional locus
*CHST8*	rs11882140	FEV1, MEF25, MEF50	Additional locus
*AGBL1*	rs78119909, rs9708220	FEV1, MEF50	Additional locus
*CSMD1*	rs13254027	MEF50	Additional locus
*CSGALNACT1*	rs142306493	MEF75	Additional locus
*AXIN1*	rs577184980	MEF75	Additional locus

## Data Availability

The genotype data generated and analysed during this study are publicly available in the Open Science Framework (OSF) repository at https://doi.org/10.17605/OSF.IO/AKQJP.

## Supplementary Materials

The following supporting information can be downloaded at: https://www.mdpi.com/article/10.3390/genes17091021/s1. Table S1: Detailed demographic, occupational, clinical and spirometric characteristics of the study participants; Table S2: Suggestive association signals (*p* < 1 × 10^−5^) identified using directly genotyped variants; Table S3: Lead suggestive association signals (*p* < 1 × 10^−5^) identified after genotype imputation.

## Abbreviations

The following abbreviations are used in this manuscript:

ATS/ERS	American Thoracic Society/European Respiratory Society
BMI	Body Mass Index
COPD	Chronic Obstructive Pulmonary Disease
CWP	Coal workers’ pneumoconiosis
FEV1	Forced Expiratory Volume in One Second
FVC	Forced Vital Capacity
GWAS	Genome-Wide Association Study
IBD	Identity-by-Descent
LD	Linkage Disequilibrium
MAF	Minor Allele Frequency
MEF25	Maximal Expiratory Flow at 25% of Forced Vital Capacity
MEF50	Maximal Expiratory Flow at 50% of Forced Vital Capacity
MEF75	Maximal Expiratory Flow at 75% of Forced Vital Capacity
PCA	Principal Component Analysis
PC	Principal Component
QC	Quality Control
QQ	Quantile–Quantile
SNP	Single Nucleotide Polymorphism
VEP	Variant Effect Predictor
